# Morphological and Genetic Variation in *Strychnos madgascariensis* Poir (Loganiaceae) at Bonamanzi Game Reserve, KwaZulu-Natal, South Africa

**DOI:** 10.3390/genes17070732

**Published:** 2026-06-24

**Authors:** Luyanda A. Mbongwe, Nontuthuko R. Ntuli, Zoliswa Mbhele

**Affiliations:** Department of Botany, Faculty of Science, Agriculture and Engineering, University of Zululand, KwaDlangezwa 3886, South Africa; luyandambongwe15@gmail.com (L.A.M.); ntulir@unizulu.ac.za (N.R.N.)

**Keywords:** *Strychnos madagascariensis*, morphotypes, intraspecific variation, SSR markers, conservation genetics, climate adaptability, population structure

## Abstract

Background: *Strychnos madagascariensis* Poir (Loganiaceae) is a drought-tolerant indigenous fruit tree of East and southern Africa, valued for its food, medicinal, and socio-economic contributions to rural communities. Despite its importance as a candidate food crop, intraspecific morphological and genetic diversity had not previously been characterized, and no simple sequence repeat (SSR) markers had been developed for this species, leaving breeders and conservation planners without the basic diversity baseline needed to prioritize material for domestication. Methods: This study assessed vegetative and reproductive trait variation, variance components, and broad-sense heritability, and SSR-based genetic diversity among 27 morphologically defined *S. madagascariensis* morphotypes at Bonamanzi Game Reserve, KwaZulu-Natal, South Africa. Three trees were measured per morphotype (81 trees total), over two growing seasons. Genetic diversity was characterized in one representative tree per morphotype using seventeen newly developed SSR loci, the first such markers reported for this species, and analyzed with population structure (STRUCTURE version 2.3.4), PCA, and Nei’s genetic distance. Results: Twenty-seven morphotypes were identified based on leaf colour, shape, hairiness and size, dominated by grey (41%), elongated (59%), less hairy (48%), and medium-sized (>50–90 mm) leaves. Fruit diameter and mass showed the highest inter-morphotype variation (r = 0.949) and also the highest broad-sense heritability (H^2^ = 55.3% and 47.8%, respectively), indicating strong genetic control of these traits and their suitability as targets for selective breeding. Environmental variance exceeded genotypic variance for most traits. A total of 144 alleles were identified across 17 SSR loci (mean 4.24 alleles/locus; mean PIC = 0.31). Population structure gave a preliminary, tentative signal of two genetic clusters (K = 2) with substantial admixture, which we interpret cautiously, given the limited sampling depth. Conclusions: This is the first study to characterize intraspecific morphological variation in *S. madagascariensis* and the first to develop SSR markers for the species. The results provide a preliminary, single-site framework for conservation genetics and crop improvement that should be validated with larger, multi-site samples. Grey morphotypes GyEvH1, GyEvH2, GyEvH3, GyRlH1 and GyEH2 combined consistent fruiting performance with favourable fruit-trait values and are proposed as priority candidates for further evaluation in domestication and breeding programmes.

## 1. Introduction

*Strychnos madagascariensis* Poir., a member of the Loganiaceae family, is an important indigenous fruit tree species in sub-Saharan Africa [1,2]. In South Africa, it has attracted increasing attention as a potential new food crop due to its edible fruits and exceptional drought tolerance, enabling it to thrive in arid environments [3]. These attributes make it a candidate for climate-smart agriculture in arid and semi-arid regions, where shifting rainfall patterns and increasing temperatures place food security under pressure [3]. Beyond its ecological significance, *S. madagascariensis* provides food and medicinal resources for both local communities and wildlife, highlighting its economic and cultural value [1,4,5].

The fruits of *S. madagascariensis* are nutrient-rich and traditionally consumed as a food source, particularly during periods of scarcity. The seeds can be processed into flour for various culinary applications and are a potential source of vegetable oil, enhancing food security in rural areas [3]. Different parts of the plant, including the roots, leaves, and bark, are used in traditional medicine to treat ailments such as malaria and gastrointestinal disorders, with bioactive compounds showing antimicrobial and anti-inflammatory effects [6]. These properties position *S. madagascariensis* as a high-value indigenous species, but realizing that value through domestication, sustainable harvesting, or in situ conservation first requires knowing how much diversity exists within the species and how it is structured, information that did not exist prior to this study.

Genetic diversity underpins a species’ capacity to adapt to environmental change and is the raw material on which breeding and domestication programmes depend [7,8]. Morphological variation in fruit size, leaf colour, and canopy architecture provides visible indicators of phenotypic divergence that complement molecular data and inform the selection of superior genotypes [1,9,10,11]. For a wild, undomesticated species being considered for the first time as a crop candidate, pairing morphological screening with molecular confirmation is the standard route to identifying which phenotypic variations are also genetically distinct, rather than plastic responses to local conditions, a distinction that matters directly for both conservation prioritization and breeding strategy.

Despite this, neither the morphological variation among intraspecific morphotypes nor the genetic structure of *S. madagascariensis* populations had been characterized before the present study. Critically, no microsatellite (SSR) markers existed for this species at all. Developing and validating a working SSR panel is therefore not a routine addition to this study but its enabling first step and is reported here as a primary contribution in its own right, independent of the specific diversity estimates the panel produces.

This study addresses both gaps by evaluating vegetative and reproductive trait variation and heritability, and by developing and applying the first SSR-based genetic diversity assessment across 27 *S. madagascariensis* morphotypes at Bonamanzi Game Reserve, KwaZulu-Natal. By integrating classical morphometric methods with molecular profiling, this research aims to provide a foundation for: (1) identifying which morphotypes represent genuinely distinct conservation units, supporting in situ conservation prioritization within a single reserve and, by extension, across the species’ wider Maputaland range; (2) flagging morphotypes whose consistent fruiting and trait expression across two contrasting seasons may indicate phenotypic resilience worth following up in formal climate-response trials; (3) informing bioprospecting and value-chain development by identifying which fruit and seed traits vary most and are most heritable; and (4) nominating, on a preliminary basis, candidate genotypes for future domestication and breeding work, pending the larger multi-site validation that a single-reserve study cannot by itself provide.

## 2. Materials and Methods

### 2.1. Study Area

**Figure 1:**
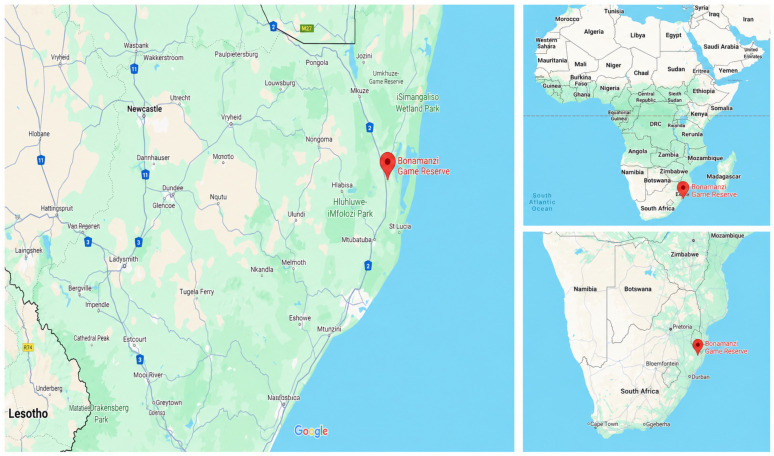
Map showing the location of Bonamanzi Game Reserve under the Big Five Municipality, KwaZulu-Natal, South Africa.

This study was conducted at Bonamanzi Game Reserve, situated in the north-eastern part of KwaZulu-Natal, South Africa, at coordinates 28°03′47.17″ S, 32°18′06.79″ E [12] (Figure 1). The reserve spans approximately 40 km^2^ [13] and lies within the savannah-biome-dominated coastal plain of northern Zululand, classified as the western Maputaland Clay Bushveld [14,15]. Forested grasslands and short forests with compound leaves constitute the dominant vegetation types [15]. Soils are deep, sandy, yellow-grey marine sands prone to leaching and low nutrient availability [12]. The climate is subtropical, with mean annual rainfall of 440–1200 mm concentrated in the summer months (October–March), and temperatures ranging from 11 °C to 35 °C [12].

### 2.2. Data Collection

#### 2.2.1. Morphotypes and Morphological Variation

In this study, a morphotype is defined as a recurring, field-recognizable combination of four discrete leaf traits such as colour, shape, hairiness and size. The morphotypes were identified during a pilot survey of the *S. madagascariensis* population at Bonamanzi Game Reserve prior to the main study. Morphotypes are descriptive phenotypic groupings used for stratified sampling and are not intended as formal taxonomic ranks; a single morphotype may, in principle, contain more than one genotype, and conversely, a single genotype could, in theory, express more than one morphotype under different environmental conditions. We treat morphotype identity as a working hypothesis about phenotypic structure that the genetic data (Section 3.10, Section 3.11, Section 3.12, Section 3.13 and Section 3.14) are then used to test, rather than as a pre-established genetic unit. Morphotypes were named according to leaf colour (greyish [Gy], pure green [G], or light green [lG]), shape (elongated [E] or roundish [R]), hairiness (very hairy [vH], hairy [H], or less hairy [lH]), and length (1 = small [≤60 mm], 2 = medium [>60–<90 mm], 3 = big [≥90 mm]) (Table 1).

Morphological traits were measured following the methodology described by Mbhele et al. [15], with modifications to sampling procedures and trait measurement to suit the objectives of this study. A stratified sampling design based on the 27 pilot-identified morphotypes was used to select 81 trees (three trees per morphotype) during the fruiting phase across a 4000-ha area of the reserve. Vegetative and reproductive data were collected over two growing seasons: season one (February–December 2023) and season two (February–December 2024).

(a) Tree and leaf characteristics

Canopy radius (m), plant height (m), and stem circumference (m) were measured using a diameter tape, telescopic height rod, and tape measure at the base, respectively. Stem diameter (m) was calculated from circumference as D = C/π, where D is diameter, C is circumference, and π = 3.14. Each tree was divided into four sections to ensure measurements were representative of the whole canopy. Leaf length and width (mm) were recorded as the mean of 12 leaves per plant from predetermined stem and branch positions [16].

(b) Fruit and seed characteristics

Fruit number per tree was counted by visual inspection from multiple angles, and twelve fruits per tree (three per cardinal direction) were harvested at the onset of ripening for measurement of diameter, mass, and pericarp thickness. Fruit yield per tree (kg/tree) was calculated from fruit number and mean fruit mass and categorized as low (≤20 kg/tree), intermediate (>20–40 kg/tree), or high (>40 kg/tree). Ten seeds per fruit were oven-dried, air-dried, de-coated, and measured for length, width, and thickness (mm) and mass (g), with total and 100-seed mass also recorded.

#### 2.2.2. Genetic Variation

A total of 27 individual trees were genotyped, one tree per morphotype, and in every case, the genotyped individual was the same tree used for the morphological measurements reported in Section 2.2.1 (one of the three morphologically measured trees per morphotype was additionally sampled for DNA). Consequently, the SSR analysis characterizes genetic variation among 27 individuals representing 27 morphotypes, not a population-level sample within each morphotype; this distinction is carried through into how population-genetic parameters (Section 2.3.2) are interpreted. The spatial distance of the sampled trees within the 4000-ha reserve was formally recorded (Table S1).

Young leaves (minimum 50 per plant) were collected from each of the 27 sampled trees, preserved in silica gel, freeze-dried, and stored at −80 °C. Genomic DNA was extracted individually from 20 mg of powdered leaf tissue per sample using a DNeasy Plant Mini Kit (QIAGEN GmbH, Hilden, Germany) following the manufacturer’s protocol, with each extraction performed in duplicate.

Genotyping used 17 SSR markers originally developed and tested on the congener *Strychnos spinosa* [17]. PCR amplification (10 µL reactions; Eppendorf Mastercycler^®^ (Eppendorf SE, Hamburg, Germany) used fluorescently labelled primers (FAM, ATTO565, ATTO550, ATTO532) under a touch-down thermal profile (initial denaturation 94 °C/2 min; 9 touch-down cycles 93 °C/15 s, 65–56 °C at −1 °C/cycle, 72 °C/30 s; 30 cycles at 55 °C annealing; final extension 72 °C/5 min). Products were resolved by capillary electrophoresis using an ABI3500 Genetic Analyser (Applied Biosystems, Thermo Fisher Scientific, Waltham, MA, USA) and scored in GeneMarker HID v2.9.5.

### 2.3. Data Analysis

#### 2.3.1. Morphological Analysis

Data were analyzed using GenStat 15th edition. Tukey’s 95% confidence interval test (*p* < 0.05) was used for mean comparisons. Pearson correlations, biplots, and Principal Component Analysis (PCA) were applied to characterize multi-trait variation. Hierarchical cluster analysis employed Ward’s method with Euclidean distance across variables.

##### Estimation of Variance Components

The phenotypic, genotypic, and environmental variances, as well as their corresponding coefficients of variation, were calculated using the method proposed by Burton and Devane [18] and later cited by Singh et al. [19] as follows:

Environmental variance ${(\delta }^{2}e)=MSE$

Genotypic variance ${(\delta }^{2}g)$

$${(\delta }^{2}e)=\frac{MSG-MSE}{r}$$

Phenotypic variance ${(\delta }^{2}p)$:

$${(\delta }^{2}p)=\;{(\delta }^{2}g)+{(\delta }^{2}e)$$

where

MSG: Square due to genotype

MSE: Square of error (environmental variance)

r: Number of replications

Phenotypic coefficient of variation (PCV) =

$$\frac{\sqrt{{(\delta }^{2}g})}{X}\times 100$$

Genotypic coefficient of variation (GCV) =

$$\frac{\sqrt{{(\delta }^{2}p})}{X}\times 100$$

where

$\delta^{2}p$: Phenotypic variation

$\delta^{2}g$: Genotypic variation

X: Grand mean of the character studied

Estimation of heritability in the broad sense: Broad-sense heritability $\left(H^{2}\right)$ was estimated as the percentage ratio of genotypic variance ${(\delta }^{2}g)$ to the phenotypic variance ${(\delta }^{2}p)$, following the formula described by Allard [20]:

$$H^{2}=\;\frac{\delta^{2}g}{\delta^{2}p}\;\times 100$$

Heritability levels were categorized as low (≤40%), moderate (>40–60%), high (>60–80%), or very high (>80%), following the criteria of Singh [19], with slight modifications. Genetic advance (GA) was calculated using the formula proposed by Allard [21] and later cited by Meena et al. [22]:

$$GA=k\times \sqrt{\frac{\delta^{2}p\;\times \delta^{2}g}{\delta^{2}p}}$$

where

$\delta^{2}p$: Phenotypic variation

$\delta^{2}g$: Genotypic variation

k: The standard selection differential at 5% selection intensity (k = 2.063)

#### 2.3.2. Genetic Analysis

Allele number, major allele frequency (MAF), gene diversity (GD), observed heterozygosity (Ho), expected heterozygosity (He), and polymorphic information content (PIC) were computed in PowerMarker version 3.25. Cervus software (version 3.0.7) was used to confirm Ho, and He estimates. Deviation from Hardy–Weinberg equilibrium (HWE) was tested per locus in Cervus using a chi-square approximation with a Bonferroni correction for multiple tests. The inbreeding coefficient (Fis) was calculated per locus as Fis = 1 − (Ho/He). The presence of null alleles was assessed for loci displaying Ho = 0.00 with He > 0 using the Brookfield [Equation (1)] estimator implemented in Micro-Checker version 2.2.3, as follows:

$$Fnull=\frac{He-Ho}{1+He}$$

where:

*Fnull*: estimated frequency of null alleles

*Ho*: observed heterozygosity

*He*: expected heterozygosity

Nei’s genetic distance was computed in PowerMarker version 3.25. Population structure was analyzed in STRUCTURE version 2.3.4 (K = 1–10, 10 runs per K, 100,000 burn-in, 100,000 MCMC iterations) and the optimal K was determined by the Evanno ΔK method via StructureSelector. Principal coordinate analysis (PCoA) was performed in GenAlEx version 6.4; dendrogram analysis in XLSTAT version 2022.1.

## 3. Results

### 3.1. Leaf Attributes and Morphotype Identification

A total of 27 morphotypes of *S. madagascariensis* were identified based on leaf traits (Table 1). Grey was the dominant leaf colour (41%), followed by green (37%) and light green (22%). Leaves were predominantly elongated (59%) over roundish (41%). Texture varied from less hairy (48%) to hairy (41%) and very hairy (11%). Medium-sized leaves were most common (44%), followed by small (41%) and big (15%) leaves.

### 3.2. Canopy Radius, Plant Height, and Stem Diameter

Canopy radius ranged from 2.90 m (GRH3) to 7.72 m (GyEvH3) across morphotypes (Table 2). The majority (66.7%) had medium-sized canopies; 18.5% were large and 14.8% small. Tree heights ranged from 2.55 m (GRH3) to 7.00 m (GRlH3), with most morphotypes (77.8%) classified as medium height. Stem diameter ranged from 0.96 m (lGRlH1) to 5.41 m (GyEvH3), with GyEvH3 significantly exceeding all other morphotypes (*p* < 0.05). GyEvH3 also had the widest canopy (7.72 m), the tallest associated morphotype class, and the thickest stems, making it the architecturally dominant morphotype.

### 3.3. Leaf Sizes

Leaf length ranged from 34 mm (lGRlH1) to 100 mm (GyEvH3) (Table 2). Most leaves were medium length (51.9%), followed by short (40.7%) and long (7.4%). Leaf width ranged from 21 mm (GyEH1) to 50 mm (GRlH3), with most being moderate (70.4%), narrow (25.9%), or wide (3.7%). GRlH3 was the only morphotype with the widest leaves, significantly different from all others (*p* < 0.05). High variation in leaf dimensions was observed within grey and green morphotypes, while light green morphotypes showed lower variation.

### 3.4. Fruit Number and Yield

**Figure 2:**
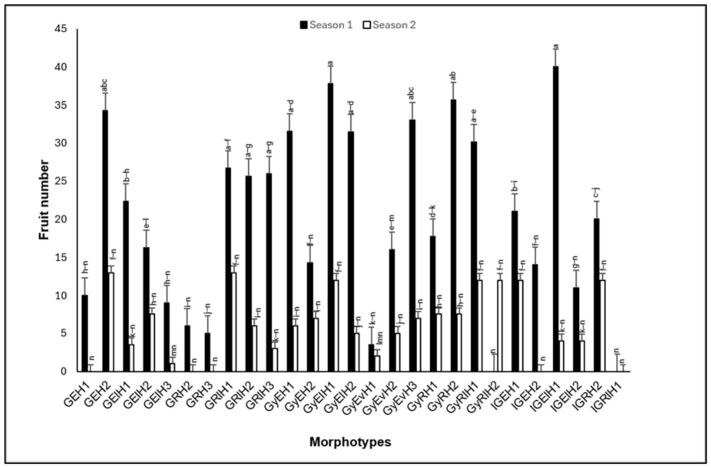
The number of fruits in *S. madagascariensis* morphotypes. Different letter(s) on the bars indicate significant differences between morphotypes and seasons as analyzed according to Tukey’s Honest Significant Difference (*p* < 0.05). Morphotypes are described in Table 1.

The majority of morphotypes (78%) produced fruits through to maturity in both seasons; only GEH1, GRH2, GRH3, GyRlH2, and lGEH2 fruited in only one season, and lGRlH1 produced no fruits in either season (Figure 2). Within season one, lGElH1 (40 fruits) and GyElH1 (38 fruits) were the highest producers. Fruit number generally declined in season two, though GyRlH2, absent from season one, is ranked among the highest in season two. GEH2 and GRlH1 (13 fruits each) led season two production.

**Figure 3:**
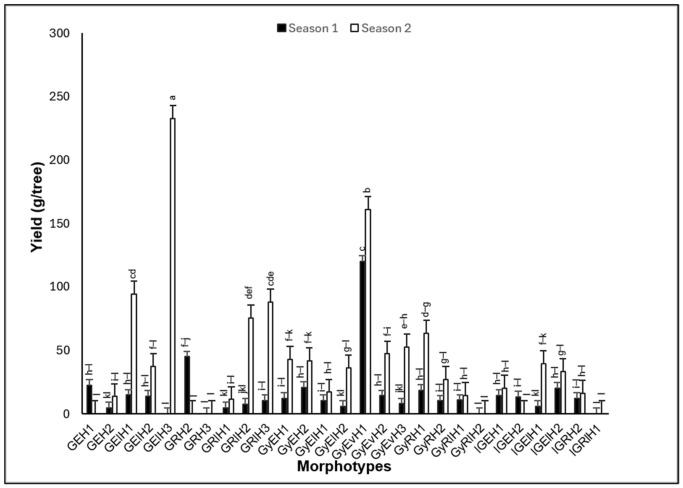
Fruit yield per morphotypes of *S. madagascariensis*. Different letter(s) on the bars indicate significant differences between morphotypes as analyzed according to Tukey’s Honest Significant Difference (*p* < 0.05). Morphotypes are described in Table 1.

Despite lower fruit numbers in season two, fruit yield was highest in season two overall. GyEvH1 (120.05 kg/tree) and GElH3 (233.00 kg/tree) were the highest yielders in seasons one and two, respectively (Figure 3). The proportion of high-yielding morphotypes (>40 kg/tree) increased from 7% in season one to 37% in season two, consistent with a biennial pattern of yield investment.

### 3.5. Fruit and Seed Characteristics

Fruit diameter ranged from 61.97 mm (GRlH1, season one) to 91.92 mm (GyElH1, season one) and from 61.35 mm (lGElH2, season two) to 86.13 mm (GyEvH3, season two) (Table 3). Large fruits dominated the population in both seasons. Fruit mass ranged from 114.6 g (GRlH1, season one) to 362.12 g (GyElH1, season one). Pericarp thickness ranged from 2.87 mm (GyRH2) to 6.24 mm (GyEH1) in season one. Morphotypes GyElH1, GyEvH1, GyRH1, and GyEvH3 consistently produced the heaviest, largest-diameter fruits across both seasons.

Seed number ranged from 10 (GRlH1 and lGElH2, season two) to 29 (GyEvH3, season two) (Table 4). Seed length ranged from 15.23 mm (GyEH1, season one) to 25.98 mm (lGEH1, season two); seed width from 14.21 mm (GyEH1) to 21.33 mm (lGEH1, season two); seed thickness from 4.92 mm (GRlH3) to 8.64 mm (GyEvH1, season two). GyEvH1 produced the heaviest total seed mass (52.29 g, season one).

### 3.6. Trait Correlations

Pearson correlations among morphological traits ranged from −0.006 to 0.995 (Table 5). Fruit diameter and fruit mass were strongly correlated (r = 0.949), and both were positively correlated with pericarp thickness, seed number, seed dimensions, and total seed mass (all r > 0.6). Canopy radius correlated positively with tree height, stem diameter, fruit diameter, pericarp thickness, seed number, seed length, and total seed mass. Leaf length and width were correlated with each other (r = 0.881) but showed weak or negligible correlations with most fruit and seed traits, suggesting that foliar and reproductive traits are governed by partially independent developmental pathways.

### 3.7. Principal Component Analysis (PCA)

**Figure 4:**
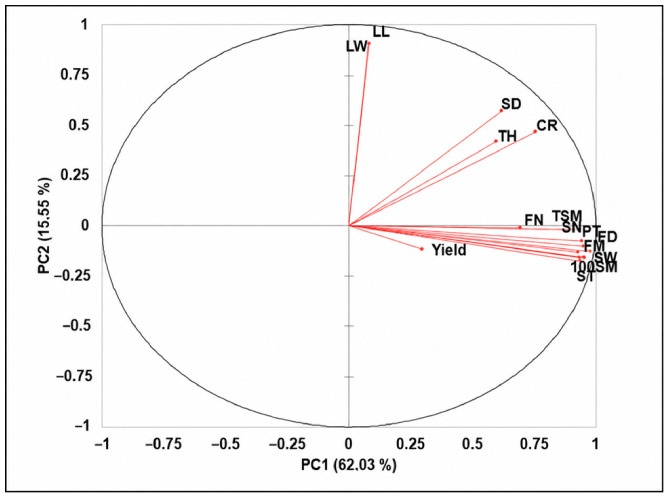
Scatter plots based on the first two principal components (PCs) for morphological traits of *S. madagascariensis*.

**Figure 5:**
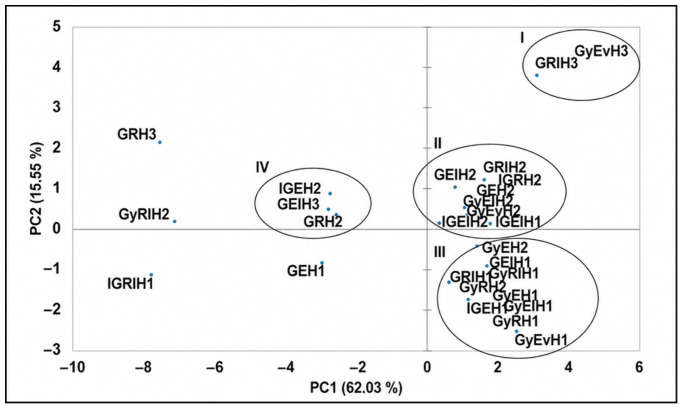
Scatter plots based on the first two principal components (PCs) for morphotypes of *S. madagascariensis.* Morphotypes are described in Table 1.

The first five principal components (PC1–PC5) explained 94.02% of cumulative morphological variance (Table 6). PC1 (62.03%) was dominated by canopy radius, stem diameter, fruit number, fruit diameter, fruit mass, pericarp thickness, seed number, seed dimensions, and seed mass, reflecting a coordinated axis of vegetative vigour and reproductive investment. PC2 (15.55%) was associated primarily with leaf length and width, indicating that foliar morphology contributes secondary, independent discriminatory power. PC3 (9.56%) was driven by fruit yield per tree. PC4 and PC5 explained 3.89% and 3.00%, respectively, with no variable loading above 0.6. Scatter plot analysis of PC1 versus PC2 identified four morphotype clusters, with the majority of fruit and seed traits loading positively on PC1, while leaf traits loaded primarily on PC2 (Figure 4). GyEvH3, GRlH3, and associated morphotypes occupied positive extremes of PC1, consistent with their large fruit and canopy dimensions (Figure 5).

### 3.8. Hierarchical Cluster Analysis

**Figure 6:**
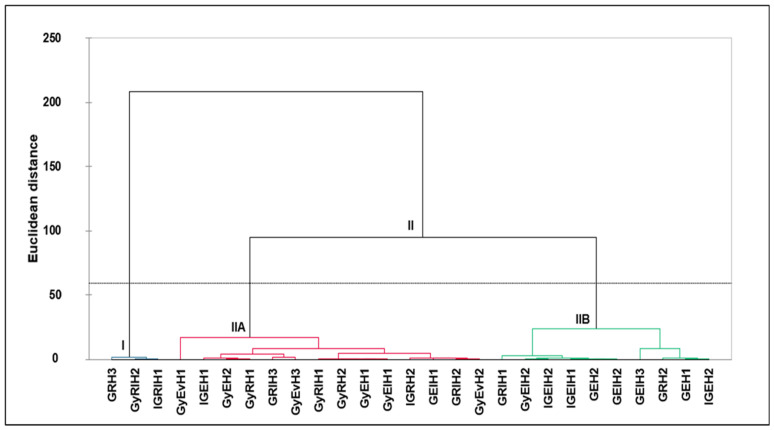
Hierarchical cluster of *S. madagascariensis* morphotypes based on Euclidean distances. Morphotypes are described in Table 1.

Ward’s hierarchical clustering based on Euclidean distance produced two major clusters (Figure 6). Cluster I comprised three non-fruiting morphotypes (GRH3, GyRlH2, lGRlH1), characterized by roundish leaves and predominantly less hairy surfaces. Cluster II is subdivided into two sub-clusters: Sub-cluster IIA (14 morphotypes: GElH1, GRlH2, GRlH3, GyEH1, GyEH2, GyElH1, GyEvH1, GyEvH2, GyEvH3, GyRH1, GyRH2, GyRlH1, lGEH1, lGRH2) representing consistently fruiting morphotypes with grey, elongated, and hairy or very hairy leaves producing heavy, large-diameter, thick-pericarp fruits. Sub-cluster IIB (10 morphotypes: GEH1, GEH2, GElH2, GElH3, GRH2, GRlH1, GyElH2, lGEH2, lGElH1, lGElH2) represented seasonally fruiting morphotypes with green, elongated, less hairy leaves producing lighter, smaller, thinner-pericarp fruits with comparatively heavier seeds. These contrasting trait constellations suggest two distinct resource allocation strategies: per-fruit investment (IIA) versus seed robustness investment (IIB).

### 3.9. Variance Components and Heritability

Environmental variance (δ^2^e) exceeded genotypic variance (δ^2^g) for all traits, indicating that phenotypic variation is predominantly shaped by environmental factors (Table 7). Phenotypic coefficients of variation (PCV) exceeded genotypic coefficients (GCV) in all cases, yet the non-trivial GCV values confirm a residual genetic contribution to trait expression. Heritability estimates were low (≤40%) for most traits. Fruit diameter showed the highest broad-sense heritability (H^2^ = 55.3%), followed by fruit mass (47.8%) and 100-seed mass (19.1%), confirming that these fruit traits are more strongly influenced by genetic factors than other measured traits and are the most suitable targets for selection. The heritability estimates reported here are derived from a single site over two seasons and should be interpreted as preliminary; formal multi-site trials are needed to partition genotype-by-environment interaction from true genetic variance.

### 3.10. Genetic Variability Among S. madagascariensis Morphotypes

#### 3.10.1. Allelic Diversity

Seventeen SSR loci were successfully amplified across all 27 *S. madagascariensis* morphotypes (*n* = 27), representing one individual per morphotype, yielding a total of 144 alleles (Table 8). This represents the first molecular characterization of genetic diversity in this species using microsatellite markers. Allele sizes ranged from 140 bp (Ssp_13) to 386 bp (Ssp_16), with a mean of 248 bp across the panel.

The number of alleles per locus ranged from 1 to 9, with a mean of 4.24 across all 17 loci. Two loci, Ssp_3 and Ssp_10, were entirely monomorphic (AN = 1, MAF = 1.00, GD = 0.00), contributing no discriminatory power. Excluding these monomorphic loci, the mean allele number among the 15 polymorphic loci rises to 5.67 per locus. The most allele-rich loci were Ssp_15 (AN = 9) and Ssp_5 (AN = 8). Major allele frequency (MAF) across all 17 loci ranged from 0.33 (Ssp_4) to 1.00 (Ssp_3 and Ssp_10), with an overall mean of 0.78.

#### 3.10.2. Gene Diversity and Polymorphic Information Content

Gene diversity (GD) ranged from 0.00 (monomorphic loci) to 0.76 (Ssp_4), with a mean of 0.33 across all loci and 0.41 among polymorphic loci (Table 8). Polymorphic information content (PIC) ranged from 0.00 to 0.72 (Ssp_4), with an overall mean of 0.31 and a polymorphic locus mean of 0.42. Four loci were highly informative (PIC ≥ 0.50): Ssp_4 (0.72), Ssp_15 (0.71), Ssp_5 (0.70), and Ssp_7 (0.63). Six loci were moderately informative (PIC 0.25–0.49). These four highly informative loci are identified as priority markers for future population genetic studies and germplasm fingerprinting.

#### 3.10.3. Observed and Expected Heterozygosity, and Inbreeding

Observed heterozygosity (Ho) ranged from 0.00 to 0.96, with an overall mean of 0.26. Expected heterozygosity (He) ranged from 0.00 to 0.83, with an overall mean of 0.54 (Table 8). In the majority of polymorphic loci, Ho was below He. The population-level Fis calculated from polymorphic loci was 0.52. However, this value should be interpreted with caution: nine loci were flagged for probable null alleles using the Brookfield Equation (1) estimator in Micro-Checker, with null allele frequency estimates ranging from 0.06 (Ssp_8) to 0.48 (Ssp_15). Null alleles cause heterozygous individuals to appear homozygous, systematically inflating apparent Fis. Among the nine loci showing no evidence of null alleles, Ho can exceed He (e.g., Ssp_1: Ho = 0.89, He = 0.64; Ssp_11: Ho = 0.96, He = 0.54), suggesting genuine heterozygote excess at these loci. The true biological Fis of the population, estimated from null-allele-free loci only, is substantially lower and may approach the Hardy–Weinberg equilibrium. A Wahlund effect arising from pooling two genetically differentiated sub-populations (Fst = 0.38) may also contribute to the apparent heterozygote deficit. Biological inbreeding cannot be reliably quantified until null allele issues are resolved through primer redesign.

### 3.11. Hardy–Weinberg Equilibrium

Per-locus HWE testing (chi-square with Bonferroni correction) identified significant departure from equilibrium at eight loci: Ssp_4 (*p* < 0.01), Ssp_5 (*p* < 0.05), Ssp_8 (*p* < 0.05), Ssp_9 (*p* < 0.01), Ssp_13 (*p* < 0.01), Ssp_14 (*p* < 0.01), Ssp_15 (*p* < 0.01), and Ssp_19 (*p* < 0.01) (Table 8). All departures were in the direction of heterozygote deficiency, consistent with null allele artifacts rather than biological inbreeding. Seven of the eight loci showing HWE departure are co-flagged for suspected null alleles. The two monomorphic loci (Ssp_3 and Ssp_10) were not testable for HWE.

### 3.12. Genetic Distance Among Morphotypes

Nei’s genetic distance among the 27 morphotypes ranged from 0.00 to 1.45 (Table S2). The greatest genetic divergence (GD = 1.45) was between morphotypes GyRlH1 and GEH2. The most genetically similar morphotypes (GD = 0.00) were GRH2 and GyRlH2, GRH3 with lGEH1 and GyEvH2. The majority of pairwise distances fell below 1.0, reflecting a baseline of moderate genetic similarity consistent with gene flow within the reserve.

### 3.13. Population Structure

**Figure 7:**
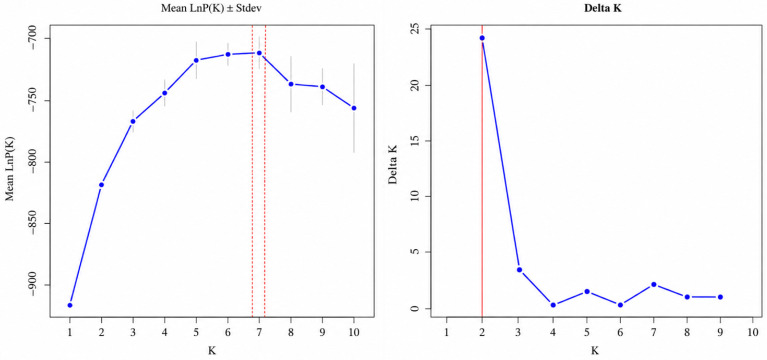
Evanno test showing plot parameters of LnP (K) and Delta K against 27 *S. madagascariensis* morphotypes.

**Figure 8:**
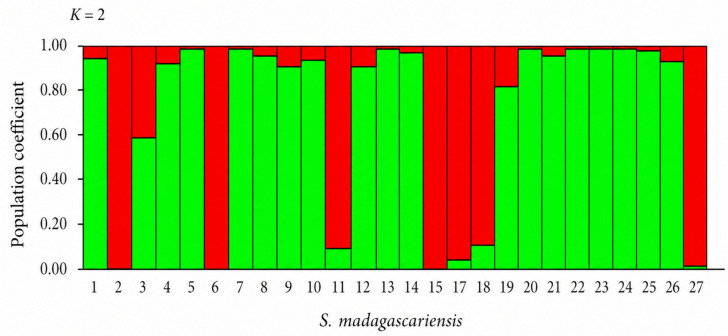
Population structure of 27 *S. madagascariensis* morphotypes revealed by SSR analysis, K = 2, K2.1 (Red), K2.2 (Green). Morphotype 1 = GElH3, 2 = GyRH1, 3 = GyEH1, 4 = GRlH2, 5 = GyEH2, 6 = GEH2, 7 = lGEH1, 8 = GRlH3, 9 = GyEvH1, 10 = GRlH1, 11 = GyEvH3, 12 = lGElH2, 13 = GyElH2, 14 = GyRlH2, 15 = lGRH2, 16 = GyRlH1, 17 = lGRlH1, 18 = GyRH2, 19 = lGEH2, 20 = GyEvH2, 21 = lGElH1, 22 = GElH2, 23 = GRH3, 24 = GRH2, 25 = GyElH1, 26 = GElH1, 27 = GEH1. Morphotypes are described in Table 1.

The Evanno ΔK method identified K = 2 as the optimal number of genetic clusters (Figure 7). Sub-population K2.1 (red) comprised morphotypes GyRH1, GEH2, and GyRlH1; sub-population K2.2 (green) included GyEH2, lGEH1, GyRlH2, GyEvH2, GElH2, GRH3, and GRH2 (Figure 8). The remaining 17 morphotypes showed admixed ancestry. Pairwise Fst between K2.1 and K2.2 was 0.38 (95% CI: 0.29–0.47), indicating substantial but incomplete differentiation between the two clusters. Given that sub-population K2.1 comprises only three morphotypes (individuals), this Fst estimate carries wide uncertainty and should be interpreted qualitatively as evidence of differentiation rather than as a precise parameter estimate. Therefore, these results (K = 2) are treated as a preliminary signal rather than a definitive population structure.

### 3.14. Principal Coordinate and Phylogenetic Analysis

**Figure 9:**
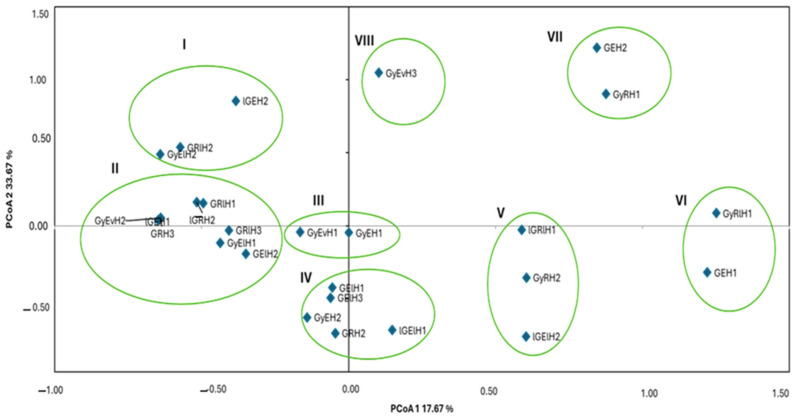
Principal coordinate analysis of *S. madagascariensis* morphotypes from SSR markers based on the genetic distance. Morphotypes are described in Table 1.

**Figure 10:**
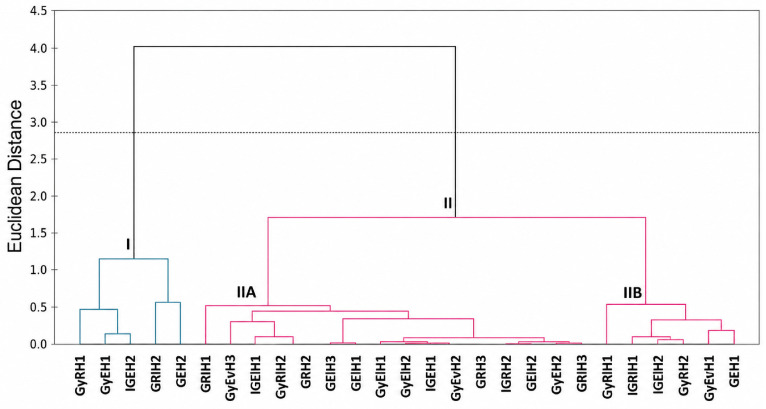
Phylogenetic relationship among *S. madagascariensis* morphotypes. Morphotypes are described in Table 1.

The first two PCoA axes accounted for 51.34% of total molecular variance and resolved eight genetic sub-clusters across four quadrants (Figure 9). Morphotypes with the smallest genetic distances clustered tightly in quadrant 1, consistent with their K2.2 sub-population assignment. The Euclidean-distance dendrogram confirmed two major phylogenetic clusters: Cluster I (non-fruiting morphotypes) and Cluster II (fruiting morphotypes), with Cluster II further subdividing into IIA and IIB as described in Figure 10.

## 4. Discussion

### 4.1. Morphological Variation and Morphotype Identification

Morphological variation in *S. madagascariensis* revealed a broad diversity of leaf traits, enabling the identification of 27 morphotypes dominated by grey, elongated, less hairy, medium-sized leaves (Table 1). Phenotypic assessment reflects the interaction of genotypic and environmental factors [23], and the pronounced intraspecific variation in colour, shape, texture, and size observed here is consistent with prior descriptions of *S. madagascariensis* displaying glossy blue to evergreen leaf coloration and elliptic to obovate shapes [5,6,9,24]. Differences in leaf shape are influenced by lamina development, which is governed by both genetic and environmental factors [25], and by biotic conditions such as local species richness [26].

Canopy radius (2.90–7.72 m) aligned closely with values previously documented at Umhlabuyalingana (2.71–10.0 m) [5]. Plant height (2.55–7.00 m) similarly corresponded to the range reported in Umhlabuyalingana (2.66–6.20 m) [5], though substantially less than the 15 m reported in Fort Hare [6], suggesting that maximum height varies across the species’ distribution. Stem diameter ranged more broadly (0.96–5.41 m) than in Umhlabuyalingana (0.02–0.35 m) [5], likely reflecting differences in resource distribution, growth strategies, and local environmental conditions [27,28].

### 4.2. Fruit Production, Yield, and Size

Fruit production at Bonamanzi (3–40 fruits/tree; 4.86–233 kg/tree) was lower in number but comparable in mass to populations in Zimbabwe (300–700 fruits/tree; 40–100 kg/tree) [29]. This pattern may reflect hotter environments promoting greater numbers of smaller, lighter fruits [30]. The highest-yielding morphotypes (GyEvH1 and GElH3) maintained high output across seasons, while low-yielding morphotypes persisted across both seasons, suggesting a heritable component to yield capacity consistent with the moderate heritability of yield-related traits reported here.

Fruit diameter (61.35–91.92 mm) and mass (114.6–362.12 g) fell within ranges documented elsewhere in the species [31,32]. Pericarp thickness varied from 2.87 to 6.24 mm; morphotypes with thinner pericarps may offer advantages in post-harvest processing efficiency without necessarily compromising nutritional quality [33]. Fruit and seed characteristics were strongly intercorrelated (Table 5), and their high loadings on PC1 confirm them as the primary axes of morphotype differentiation.

### 4.3. Variance Components, Heritability, and Climate Adaptability

The predominance of environmental over genotypic variance for most morphological traits reflects a high degree of phenotypic plasticity in *S. madagascariensis,* a feature that is ecologically advantageous in the variable, semi-arid environments the species inhabits. Phenotypic plasticity enables individuals to modulate growth, leaf, and reproductive traits in response to seasonal drought, soil nutrient variability, and interannual rainfall fluctuations [34].

Morphotypes such as GyEvH3, GyEvH1, and GyRlH1, which maintained consistent fruiting and canopy expansion across both contrasting seasons of this study, demonstrate a degree of phenotypic stability that may be relevant for identifying resilient genotypes. However, these two seasons represent a single site and a limited environmental range; formal phenological studies manipulating water availability and temperature will be necessary to quantify climate adaptability.

Fruit diameter (H^2^ = 55.3%) and fruit mass (H^2^ = 47.8%) showed substantially higher heritability than other traits, indicating that these parameters are more strongly influenced by genetic factors than by the environment. These findings have direct implications for breeding: phenotypic pre-selection for fruit size is likely to be effective, while traits with low heritability will require larger populations, replicated multi-site trials, and genomic selection approaches to achieve reliable genetic gain. It bears emphasis that these heritability estimates derive from a single site over two seasons and should be treated as preliminary pending multi-environment validation.

### 4.4. SSR Genetic Diversity and Population Structure

#### 4.4.1. Allelic Richness in Comparative Context

The 17 SSR markers developed for *S. madagascariensis* yielded a total of 144 alleles (mean AN = 4.24), which is broadly comparable to values reported for the congeneric *S. spinosa* using 14 markers in an overlapping geographic region (159 alleles; mean AN = 5.68) [17]. Among polymorphic loci only, the mean AN of 5.67 in *S. madagascariensis* approaches the *S. spinosa* value more closely, suggesting that the underlying allelic richness of the two species is comparable when monomorphic loci are excluded.

Relative to SSR studies in other woody plant species, allelic diversity in *S. madagascariensis* falls within the moderate range. Studies of cultivated and wild fruit trees such as *Prunus avium* (mean AN = 11.8) [35], *Psidium guajava* (mean AN = 8.6) [36], and *Jatropha curcas* (mean AN = 3.5–8.2) [37] typically report higher mean allele numbers per locus. It should be noted that cultivated species carry signatures of human-mediated gene flow across wide geographic ranges, making them imperfect comparators for a wild, spatially constrained population such as this one. The comparatively modest allelic richness in *S. madagascariensis* is consistent with a wild, uncultivated population in a 40 km^2^ reserve with potentially limited external gene flow, and falls within ranges reported for other wild, geographically restricted tree populations. Broader geographic sampling across KwaZulu-Natal and Mozambique is necessary to fully characterize species-level allelic diversity.

#### 4.4.2. Polymorphic Information Content and Marker Utility

The mean PIC of 0.31 across all 17 loci (0.42 among polymorphic loci) is lower than that reported for *S. spinosa* (PIC = 0.57) [17] and for cultivated species such as *P. avium* (PIC = 0.64) [35]. The inclusion of two monomorphic loci (PIC = 0.00 each) suppresses the overall mean, and high null allele frequencies at Ssp_4 and Ssp_15 may partially obscure true informativeness by masking heterozygous genotypes. The genuinely informative portion of the panel, the four loci with PIC > 0.50 (Ssp_4, Ssp_5, Ssp_7, Ssp_15) and six with PIC 0.25–0.49, represents a core of ten markers with adequate to high discriminatory power sufficient for population structure analysis, morphotype differentiation, and parentage testing at the scale of this study. The two monomorphic loci (Ssp_3, Ssp_10) should be excluded from population structure and diversity analyses and redesigned or replaced in future work.

#### 4.4.3. Heterozygote Deficit, Inbreeding, and Null Alleles

The population-level Fis of 0.52 must be interpreted primarily as a technical artifact rather than evidence of biological inbreeding. Nine loci were flagged for probable null alleles, with estimates up to 0.48 at Ssp_15. All eight loci departing from HWE do so in the direction of heterozygote deficiency, and all eight are co-flagged for null alleles, a pattern consistent with null alleles as the proximate driver. Among the nine null-allele-free loci, the estimated Fis is substantially lower (approximately −10 to +15%), suggesting the population may be close to Hardy–Weinberg equilibrium in the absence of artifacts. An additional contribution from the Wahlund effect cannot be excluded, given the pairwise Fst = 0.38 between sub-populations; pooling genetically differentiated groups inflates apparent heterozygote deficits even without true inbreeding. Resolving null-allele issues through primer redesign and genotyping sub-populations separately remains an important priority for future work.

### 4.5. Conservation and Breeding Implications

For breeding and domestication, the high-PIC markers Ssp_4, Ssp_5, Ssp_7, and Ssp_15 are recommended as a core fingerprinting panel for verifying morphotype identity, confirming the genetic distinctiveness of putative superior morphotypes, monitoring the genetic integrity of cultivated material, and parentage assignment in controlled pollination experiments. Strategic crossing designs that cross K2.1 morphotypes (GyRH1, GEH2, GyRlH1) with K2.2 morphotypes (GyEH2, lGEH1, GRH2) would maximize heterozygosity in progeny by exploiting complementary allele pools.

Given that fruit diameter and mass show the highest heritability and can be reliably assessed in the field, a combined strategy of phenotypic pre-selection for fruit size followed by molecular verification using the four high-PIC SSR markers would represent the most efficient approach to domestication programme development. The wide genetic variation documented within a single reserve strongly supports the hypothesis that KwaZulu-Natal represents a centre of diversity for *S. madagascariensis*, with direct policy implications for prioritizing in situ conservation and broader surveys across the Maputaland region and into southern Mozambique.

### 4.6. Limitations

Several limitations of this study warrant explicit acknowledgement. First, because each morphotype is represented by a single genotyped individual, the SSR analysis reflects individual-level rather than population-level allele frequencies in the strict sense; conclusions about population genetic parameters (Fis, Fst, HWE) should be interpreted accordingly and validated with larger sample sizes in future studies. Second, heritability estimates derive from a single site over two seasons and are susceptible to confounding by genotype-by-environment interaction; multi-site trials are needed before these values are used in formal breeding programmes. Third, null alleles were detected at nine of 17 loci, inflating the apparent inbreeding coefficient and potentially biasing diversity estimates; primer redesign at affected loci is a prerequisite for definitive population genetic inference. Fourth, the pairwise Fst of 0.38 between K2.1 and K2.2 sub-populations should be treated as an indicative estimate given that K2.1 comprises only three individuals. Addressing these limitations through expanded geographic sampling, independent SSR loci, and replicated field trials will substantially strengthen the conclusions presented here.

## 5. Conclusions

This study provides the first integrated characterization of morphological and genetic diversity in *S. madagascariensis*, addressing a critical knowledge gap for this overlooked indigenous crop species. Twenty-seven morphotypes were identified at Bonamanzi Game Reserve based on leaf traits, showing broad variation in vegetative architecture, fruit production, and seed characteristics across two growing seasons.

The primary discriminating factors were canopy radius, stem diameter, and fruit and seed traits (PC1, 62.03%), with foliar morphology contributing secondary variation (PC2, 15.55%). Hierarchical clustering grouped morphotypes into non-fruiting (Cluster I), consistently fruiting (Sub-cluster IIA), and seasonally fruiting (Sub-cluster IIB) groups, each reflecting a distinct reproductive strategy. Environmental variance predominated over genotypic variance for most traits; however, fruit diameter (H^2^ = 55.3%) and fruit mass (H^2^ = 47.8%) showed sufficiently high heritability to be viable preliminary targets for selective breeding, pending multi-site validation.

This is the first study to develop SSR markers for *S. madagascariensis* and apply them to genetic analysis. Seventeen markers amplified 144 alleles, resolving two genetic sub-populations (Fst = 0.38) within the reserve. A population-level Fis of 0.52 is interpreted primarily as a technical artifact driven by null alleles at nine loci and a possible Wahlund effect; it should not be taken as evidence of biological inbreeding without further investigation. The genetic diversity documented within a single, geographically compact reserve highlights the potential of KwaZulu-Natal as a centre of diversity for the species.

Grey morphotypes GyEvH1, GyEvH2, GyEvH3, GyRlH1, and GyEH2 are recommended as priority candidates for domestication and breeding programmes, based on their superior morphological performance, consistent fruiting behaviour, and genetic distinctiveness. Future work should expand sampling across the broader distribution range of *S. madagascariensis* in KwaZulu-Natal and Mozambique, redesign primers at null-allele-affected loci, conduct controlled phenological trials to formally quantify climate adaptability, and deposit SSR genotype data in a public repository to support the conservation genetics community.

## Figures and Tables

**Table 1:** Plant traits used to name *S. madagascariensis* morphotypes.

Morphotype	Plant Traits				
	Leaf Colour	Leaf Shape	Leaf Hairiness	Leaf Size	Leaf Size Class
GEH1	Green	Elongated	Hairy	Small	1
GEH2	Green	Elongated	Hairy	Medium	2
GElH1	Green	Elongated	Less hairy	Small	1
GElH2	Green	Elongated	Less hairy	Medium	2
GElH3	Green	Elongated	Less hairy	Big	3
GRH2	Green	Roundish	Hairy	Medium	2
GRH3	Green	Roundish	Hairy	Big	3
GRlH1	Green	Roundish	Less hairy	Small	1
GRlH2	Green	Roundish	Less hairy	Medium	2
GRlH3	Green	Roundish	Less hairy	Big	3
GyEH1	Grey	Elongated	Hairy	Small	1
GyEH2	Grey	Elongated	Hairy	Medium	2
GyElH1	Grey	Elongated	Less hairy	Small	1
GyElH2	Grey	Elongated	Less hairy	Medium	2
GyEvH1	Grey	Elongated	Very hairy	Small	1
GyEvH2	Grey	Elongated	Very hairy	Medium	2
GyEvH3	Grey	Elongated	Very hairy	Big	3
GyRH1	Grey	Roundish	Hairy	Small	1
GyRH2	Grey	Roundish	Hairy	Medium	2
GyRlH1	Grey	Roundish	Less hairy	Small	1
GyRlH2	Grey	Roundish	Less hairy	Medium	2
lGEH1	Light green	Elongated	Hairy	Small	1
lGEH2	Light green	Elongated	Hairy	Medium	2
lGElH1	Light green	Elongated	Less hairy	Small	1
lGElH2	Light green	Elongated	Less hairy	Medium	2
lGRH2	Light green	Roundish	Hairy	Medium	2
lGRlH1	Light green	Roundish	Less hairy	Small	1

**Table 2:** Variation in the vegetative traits of *S. madagascariensis* morphotypes.

Morphotype	Plant Traits			Leaf Traits	
	CR	TH	SD	LL	LW
GEH1	4.40 ^d–i^	5.00 ^b–e^	1.69 ^e–h^	44 ^j–m^	22 ^kL^
GEH2	6.44 ^abc^	4.80 ^b–e^	3.27 ^b^	66 ^c–f^	27 ^f–l^
GElH1	5.73 ^b–e^	4.63 ^c–f^	2.49 ^b–g^	44 ^j–m^	28 ^f–l^
GElH2	5.42 ^c–g^	4.84 ^b–e^	2.97 ^b–e^	69 ^cde^	36 ^b–e^
GElH3	4.29 ^e–i^	3.55 ^f–i^	1.93 ^c–h^	75 ^c^	33 ^d–g^
GRH2	4.65 ^d–i^	4.30 ^d–g^	2.10 ^b–h^	63 ^d–g^	30 ^e–i^
GRH3	2.90 ^i^	2.55 ^i^	1.49 ^fgh^	88 ^b^	43 ^b^
GRlH1	4.98 ^c–g^	4.17 ^e–h^	3.19 ^bc^	36 ^mn^	23 ^i–l^
GRlH2	6.05 ^a–d^	4.50 ^c–g^	2.65 ^b–g^	71 ^cd^	40 ^bcd^
GRlH3	7.30 ^ab^	7.00 ^a^	3.03 ^bcd^	93 ^ab^	50 ^a^
GyEH1	5.31 ^c–g^	4.85 ^b–e^	2.51 ^b–g^	43 ^j–m^	21 ^L^
GyEH2	4.60 ^d–i^	4.00 ^e–h^	2.17 ^b–h^	64 ^d–g^	34 ^c–f^
GyElH1	5.37 ^c–g^	4.90 ^b–e^	2.21 ^b–h^	40 ^k–n^	23 ^jkl^
GyElH2	5.76 ^b–e^	5.10 ^b–e^	2.17 ^b–h^	70 ^cde^	32 ^e–h^
GyEvH1	4.67 ^d–h^	4.10 ^e–h^	1.53 ^fgh^	45 ^j–m^	24 ^i–l^
GyEvH2	4.73 ^c–h^	6.00 ^ab^	2.26 ^b–h^	64 ^d–g^	33 ^efg^
GyEvH3	7.72 ^a^	6.00 ^ab^	5.41 ^a^	100 ^a^	41 ^bc^
GyRH1	5.62 ^b–f^	4.44 ^d–g^	1.75 ^d–h^	37 ^lmn^	22 ^kL^
GyRH2	4.18 ^e–i^	3.40 ^ghi^	1.39 ^gh^	59 ^fgh^	29 ^f–k^
GyRlH1	5.48 ^c–g^	5.38 ^bcd^	1.84 ^d–h^	41 ^k–n^	27 ^f–l^
GyRlH2	3.84 ^ghi^	4.10 ^e–h^	1.15 ^h^	49 ^ijk^	28 ^f–l^
lGEH1	3.90 ^f–i^	4.35 ^d–g^	2.12 ^b–h^	45 ^jkl^	26 ^g–l^
lGEH2	5.33 ^c–g^	5.65 ^bc^	1.83 ^d–h^	60 ^efg^	30 ^e–j^
lGElH1	6.12 ^a–d^	5.10 ^b–e^	2.90 ^b–e^	51 ^hij^	31 ^e–h^
lGElH2	5.16 ^c–g^	4.93 ^b–e^	2.26 ^b–h^	63 ^d–g^	32 ^efg^
lGRH2	5.37 ^c–g^	6.00 ^ab^	2.74 ^b–f^	55 ^ghi^	37 ^b–e^
lGRlH1	3.08 ^hi^	3.00 ^hi^	0.96 ^h^	34 ^n^	25 ^h–l^

CR, canopy radius (m); TH, tree height (m); SD, stem diameter (m); LL, leaf length (mm); LW, leaf width (mm). Mean values followed by different letter(s) within a column (for morphotypes) differ significantly at *p* < 0.05. Morphotypes are described in Table 1.

**Table 3:** Variation in the diameter, mass, and pericarp thickness of *S. madagascariensis* fruits.

Morphotype	Fruit Diameter		Fruit Mass		Pericarp Thickness	
	S1	S2	S1	S2	S1	S2
GEH1	71.02 ^f–n^	0 ^o^	217.79 ^d–l^	0 ^m^	4.29 ^d–j^	0 ^m^
GEH2	67.52 ^j–n^	67.87 ^h–n^	160.71 ^g–l^	175.21 ^e–l^	4.06 ^d–l^	3.82 ^e–l^
GElH1	79.62 ^b–i^	71.92 ^d–n^	269.67 ^a–g^	199.25 ^e–l^	5.63 ^ab^	3.83 ^e–l^
GElH2	74.74 ^b–l^	62.56 ^mn^	211.21 ^d–l^	139.71 ^jkl^	4.55 ^b–i^	3.61 ^h–l^
GElH3	0 ^o^	74.50 ^b–l^	0 ^m^	233.00 ^b–k^	0 ^m^	4.60 ^b–i^
GRH2	78.75 ^b–j^	0 ^o^	264.00 ^a–h^	0 ^m^	3.60 ^h–l^	0 ^m^
GRH3	0 ^o^	0 ^o^	0 ^m^	0 ^m^	0 ^m^	0 ^m^
GRlH1	61.97 ^n^	64.23 ^L–n^	114.6 ^lm^	147.12 ^i–l^	4.07 ^d–l^	4.41 ^c–j^
GRlH2	70.27 ^f–n^	76.57 ^b–k^	183.38 ^e–l^	253.08 ^a–j^	3.87 ^e–l^	3.58 ^h–l^
GRlH3	78.73 ^b–j^	78.67 ^b–j^	272.29 ^a–g^	255.17 ^a–j^	3.87 ^e–l^	4.07 ^d–l^
GyEH1	81.60 ^a–f^	75.28 ^b–l^	289.21 ^a–e^	248.91 ^a–j^	6.24 ^a^	4.56 ^b–i^
GyEH2	78.07 ^b–j^	79.72 ^b–h^	267.00 ^a–g^	271.88 ^a–g^	3.53 ^h–l^	3.93 ^e–l^
GyElH1	91.92 ^a^	74.72 ^b–l^	362.12 ^a^	202.71 ^e–l^	5.22 ^a–d^	5.01 ^b–e^
GyElH2	68.73 ^h–n^	67.82 ^i–n^	184.88 ^e–l^	176.33 ^e–l^	3.40 ^i–l^	3.33 ^jkl^
GyEvH1	83.10 ^a–d^	78.80 ^b–j^	343.00 ^ab^	263.25 ^a–h^	4.50 ^b–j^	3.70 ^g–l^
GyEvH2	75.10 ^b–l^	73.88 ^c–m^	225.46 ^c–l^	226.7 ^c–l^	4.21 ^d–k^	3.86 ^e–l^
GyEvH3	76.22 ^b–k^	86.13 ^ab^	259.08 ^a–i^	360.36 ^a^	4.85 ^b–g^	5.67 ^ab^
GyRH1	79.73 ^b–h^	81.00 ^a–g^	271.46 ^a–g^	283.92 ^a–f^	4.17 ^d–k^	3.88 ^e–l^
GyRH2	82.96 ^a–e^	71.13 ^e–n^	334.00 ^abc^	202.78 ^e–l^	2.87 ^L^	3.79 ^f–l^
GyRlH1	84.77 ^abc^	69.07 ^h–n^	319.25 ^a–d^	170.38 ^f–l^	5.52 ^abc^	3.62 ^h–l^
GyRlH2	0 ^o^	0 ^o^	0 ^m^	0 ^m^	0 ^m^	0 ^m^
lGEH1	78.17 ^b–j^	77.93 ^b–j^	255.75 ^a–i^	236.12 ^b–k^	4.14 ^d–k^	4.24 ^d–j^
lGEH2	68.67 ^h–n^	0 ^o^	188.44 ^e–l^	0 ^m^	4.27 ^d–j^	0 ^m^
lGElH1	75.10 ^b–l^	66.00 ^k–n^	226.5 ^c–l^	151.12 ^h–l^	5.21 ^a–d^	4.92 ^b–f^
lGElH2	69.44 ^g–n^	61.35 ^n^	187.83 ^e–l^	126.38 ^kL^	4.42 ^c–j^	3.00 ^kL^
lGRH2	78.57 ^b–j^	70.93 ^f–n^	242.08 ^b–k^	189.75 ^e–l^	4.63 ^b–h^	3.92 ^e–l^
lGRlH1	0 ^o^	0 ^o^	0 ^m^	0 ^m^	0 ^m^	0 ^m^

Fruit diameter (mm); Fruit mass (g); Pericarp thickness (mm); S1, season one; S2, season two. Mean values followed by different letter(s) within a column (for morphotypes) and a row (for seasonal variation) of each trait differ significantly at *p* < 0.05. Morphotypes are described in Table 1.

**Table 4:** Variation in seed number, mass, and sizes of *S. madagascariensis* morphotypes.

Morphotype	Seed Number		Total Seed Mass		Seed Length		Seed Width		Seed Thickness	
	S1	S2	S1	S2	S1	S2	S1	S2	S1	S2
GEH1	19 ^a–i^	0 ^j^	27.53 ^c–j^	0 ^k^	22.44 ^b–g^	0 ^q^	19.30 ^a–d^	0 ^q^	6.12 ^d–k^	0 ^L^
GEH2	16 ^c–i^	17 ^b–i^	20.43 ^f–j^	25.76 ^d–j^	22.32 ^b–h^	21.68 ^c–k^	16.92 ^e–n^	18.60 ^b–g^	5.86 ^h–k^	5.97 ^e–k^
GElH1	23 ^a–f^	13 ^f–i^	26.16 ^d–j^	25.38 ^d–j^	21.59 ^c–k^	22.20 ^b–h^	17.23 ^c–m^	18.57 ^b–g^	5.64 ^ijk^	7.64 ^abc^
GElH2	19 ^a–i^	15 ^d–i^	21.73 ^e–j^	17.48 ^hij^	19.78 ^j–o^	18.68 ^mno^	16.68 ^e–o^	15.14 ^L–p^	6.87 ^b–j^	6.97 ^b–j^
GElH3	0 ^j^	18 ^b–i^	0 ^k^	28.46 ^c–j^	0 ^q^	20.00 ^i–n^	0 ^q^	15.80 ^j–p^	0 ^L^	7.64 ^abc^
GRH2	19 ^a–i^	0 ^j^	34.23 ^b–h^	0 ^k^	22.67 ^b–f^	0 ^q^	18.98 ^b–e^	0 ^q^	8.03 ^ab^	0 ^L^
GRH3	0 ^j^	0 ^j^	0 ^k^	0 ^k^	0 ^q^	0 ^q^	0 ^q^	0 ^q^	0 ^L^	0 ^L^
GRlH1	12 ^hi^	10 ^hi^	18.46 ^f–j^	19.66 ^f–j^	19.93 ^i–o^	21.18 ^e–l^	16.22 ^h–p^	18.45 ^b–h^	7.79 ^ab^	7.21 ^a–h^
GRlH2	16 ^d–i^	23 ^a–f^	27.82 ^c–j^	34.74 ^b–h^	19.19 ^L–o^	18.51 ^mno^	15.43 ^k–p^	14.56 ^op^	7.47 ^a–d^	7.33 ^a–g^
GRlH3	19 ^a–i^	17 ^b–i^	37.75 ^a–e^	34.93 ^b–g^	19.53 ^k–o^	21.59 ^c–k^	16.48 ^g–p^	19.40 ^a–d^	4.92 ^k^	7.42 ^a–f^
GyEH1	18 ^b–i^	15 ^d–i^	13.51 ^jk^	27.30 ^c–j^	15.23 ^p^	22.66 ^b–f^	14.21 ^p^	18.17 ^b–i^	7.24 ^a–h^	7.64 ^abc^
GyEH2	15 ^d–i^	14 ^e–i^	34.96 ^b–g^	26.30 ^d–j^	23.39 ^bcd^	23.90 ^ab^	19.68 ^ab^	19.53 ^abc^	7.07 ^b–j^	6.23 ^c–k^
GyElH1	27 ^ab^	13 ^f–i^	30.40 ^c–j^	19.88 ^f–j^	20.15 ^h–n^	22.43 ^b–g^	16.57 ^f–o^	18.87 ^b–f^	7.69 ^abc^	5.79 ^h–k^
GyElH2	14 ^f–i^	14 ^d–i^	22.28 ^e–j^	25.06 ^e–j^	20.18 ^h–n^	23.63 ^bc^	16.36 ^g–p^	18.93 ^b–e^	6.06 ^d–k^	6.82 ^b–j^
GyEvH1	26 ^abc^	20 ^a–i^	52.29 ^a^	43.91 ^abc^	22.10 ^b–i^	22.07 ^b–i^	18.52 ^b–h^	18.53 ^b–g^	7.43 ^a–e^	8.64 ^a^
GyEvH2	17 ^b–i^	18 ^b–i^	25.14 ^d–j^	25.16 ^d–j^	18.53 ^mno^	17.78 ^o^	16.35 ^g–p^	15.76 ^j–p^	8.29 ^ab^	7.19 ^a–h^
GyEvH3	17 ^b–i^	29 ^a^	32.21 ^b–i^	48.81 ^ab^	18.93 ^mno^	21.36 ^d–l^	14.50 ^op^	17.43 ^b–l^	5.62 ^jk^	7.10 ^b–i^
GyRH1	24 ^a–e^	19 ^a–i^	27.64 ^c–j^	42.41 ^a–d^	18.22 ^no^	23.77 ^bc^	14.70 ^nop^	18.97 ^b–e^	5.96 ^f–k^	7.62 ^abc^
GyRH2	24 ^a–d^	17 ^b–i^	33.21 ^b–i^	25.37 ^d–j^	19.60 ^k–o^	19.80 ^j–o^	17.48 ^b–k^	17.33 ^c–l^	7.03 ^b–j^	7.34 ^a–g^
GyRlH1	20 ^a–h^	15 ^d–i^	35.33 ^a–f^	22.50 ^e–j^	23.04 ^b–e^	18.43 ^mno^	17.16 ^d–m^	14.96 ^m–p^	5.97 ^e–k^	7.02 ^b–j^
GyRlH2	0 ^j^	0 ^j^	0 ^k^	0 ^k^	0 ^q^	0 ^q^	0 ^q^	0 ^q^	0 ^L^	0 ^L^
lGEH1	15 ^d–i^	12 ^ghi^	21.45 ^e–j^	27.09 ^c–j^	19.24 ^L–o^	25.98 ^a^	15.58 ^k–p^	21.33 ^a^	7.77 ^ab^	5.94 ^g–k^
lGEH2	14 ^e–i^	0 ^j^	23.77 ^e–j^	0 ^k^	18.84 ^mno^	0 ^q^	16.03 ^i–p^	0 ^q^	7.97 ^ab^	0 ^L^
lGElH1	22 ^a–g^	12 ^hi^	26.55 ^d–j^	19.32 ^f–j^	19.64 ^k–o^	20.35 ^g–n^	14.93 ^m–p^	16.05 ^i–p^	7.99 ^ab^	7.68 ^abc^
lGElH2	15 ^d–i^	10 ^ij^	17.81 ^g–j^	16.88 ^ijk^	20.55 ^f–m^	21.83 ^b–j^	17.12 ^d–m^	18.13 ^b–i^	7.60 ^abc^	6.26 ^c–k^
lGRH2	19 ^a–i^	12 ^ghi^	31.99 ^b–i^	22.19 ^e–j^	20.60 ^f–m^	19.75 ^j–o^	17.92 ^b–j^	17.98 ^b–j^	7.10 ^b–i^	7.50 ^a–d^
lGRlH1	0 ^j^	0 ^j^	0 ^k^	0 ^k^	0 ^q^	0 ^q^	0 ^q^	0 ^q^	0 ^L^	0 ^L^

Total seed mass (g); Seed length (mm); Seed width (mm); Seed thickness (mm); S1, season one; S2, season two. Mean values followed by different letter(s) within a column (for morphotypes) and a row (for seasonal variation) of each trait differ significantly at *p* < 0.05. Morphotypes are described in Table 1.

**Table 5:** Correlation among *S. madagascariensis* morphological traits.

Variables	CR	TH	SD	LL	LW	FN	Yield	FD	FM	PT	SN	SL	SW	ST	TSM
TH	**0.768**														
SD	**0.797**	0.557													
LL	0.368	0.241	0.486												
LW	0.328	0.300	0.388	**0.881**											
FN	0.595	0.390	0.534	−0.036	−0.054										
Yield	0.111	−0.043	−0.012	0.109	0.042	−0.244									
FD	**0.643**	0.515	0.499	−0.011	−0.006	**0.688**	0.288								
FM	0.584	0.454	0.430	0.022	0.001	0.584	0.377	**0.949**							
PT	**0.693**	0.556	**0.617**	−0.064	−0.069	**0.746**	0.222	**0.946**	**0.872**						
SN	**0.681**	0.473	0.497	0.053	0.018	**0.619**	0.392	**0.942**	**0.945**	**0.882**					
SL	**0.622**	0.474	0.493	−0.038	−0.045	**0.676**	0.251	**0.972**	**0.873**	**0.917**	**0.876**				
SW	0.599	0.489	0.475	−0.037	−0.035	**0.658**	0.247	**0.972**	**0.876**	**0.908**	**0.871**	**0.995**			
ST	0.577	0.474	0.468	−0.066	−0.045	**0.640**	0.301	**0.957**	**0.853**	**0.926**	**0.883**	**0.947**	**0.951**		
TSM	**0.637**	0.457	0.454	0.125	0.116	0.450	0.514	**0.878**	**0.926**	**0.767**	**0.928**	**0.825**	**0.822**	**0.803**	
100SM	0.572	0.460	0.448	−0.027	0.000	0.540	0.360	**0.948**	**0.895**	**0.866**	**0.852**	**0.958**	**0.959**	**0.922**	**0.883**

Significant values ≥ 0.6 are in bold. CR, canopy radius; TH, tree height; SD, stem diameter; LL, leaf length; LW, leaf width; FN, fruit number; FD, Fruit diameter; FM, fruit mass; PT, pericarp thickness; SN, seed number; SL, seed length; SW, seed width; ST, seed thickness; TSM, total seed mass; 100SM, one hundred seed mass.

**Table 6:** Principal components of traits *S. madagascariensis* morphotypes.

Variables	PC1	PC2	PC3	PC4	PC5
CR	0.756	0.468	−0.177	0.273	0.184
TH	0.598	0.419	−0.254	0.552	−0.280
SD	0.618	0.569	−0.238	0.000	0.378
LL	0.084	0.903	0.270	−0.239	−0.003
LW	0.073	0.885	0.243	−0.213	−0.249
FN	0.699	−0.010	−0.560	−0.269	0.144
Yield	0.299	−0.117	0.864	0.184	0.256
FD	0.983	−0.130	0.013	−0.072	−0.077
FM	0.930	−0.131	0.155	−0.048	−0.039
PT	0.953	−0.103	−0.143	0.022	0.102
SN	0.945	−0.075	0.130	−0.012	0.074
SL	0.957	−0.158	−0.027	−0.100	−0.087
SW	0.953	−0.160	−0.017	−0.096	−0.135
ST	0.940	−0.178	0.012	−0.064	−0.076
TSM	0.897	−0.020	0.324	0.034	−0.002
100SM	0.936	−0.158	0.131	−0.049	−0.143
Eigenvalue	9.924	2.488	1.530	0.622	0.479
Variability (%)	62.026	15.548	9.561	3.889	2.995
Cumulative %	62.026	77.574	87.135	91.023	94.019

Significant values ≥ 0.6. CR, canopy radius; TH, tree height; SD, stem diameter; LL, leaf length; LW, leaf width; FN, fruit number; FD, Fruit diameter; FM, fruit mass; PT, pericarp thickness; SN, seed number; SL, seed length; SW, seed width; ST, seed thickness; TSM, total seed mass; 100SM, one hundred seed mass.

**Table 7:** Genetic parameters for morphological traits of *S. madagascariensis* morphotypes.

Variables	δ^2^g	δ^2^e	δ^2^p	GM	PCV	GCV	ECV	H^2^
CR	0.2	1.3	1.6	5.1	5.5	2.1	5.1	15.0
TH	0.1	0.6	0.7	4.7	4.0	1.5	3.7	13.9
SD	0.1	0.7	0.8	2.3	5.9	1.6	5.6	7.8
LL	5.4	36.3	41.6	58.1	8.5	3.0	7.9	12.8
LW	4.2	23.1	27.3	30.6	9.5	3.7	8.7	15.4
FN	0.8	82.8	83.6	13.0	25.4	2.5	25.3	1.0
FD	64.0	51.7	115.7	60.8	13.8	10.3	9.2	55.3
FM	4485.7	4896.0	9381.7	188.2	70.6	48.8	51.0	47.8
PT	0.0	0.5	0.6	3.5	4.1	1.1	3.9	7.0
Yield	73.6	552.3	625.9	29.3	46.2	15.8	43.4	11.8
SN	1.3	38.2	39.5	14.0	16.8	3.0	16.5	3.3
TSM	4.4	109.4	113.8	22.6	22.4	4.4	22.0	3.8
100SM	65.5	277.3	342.8	143.2	15.5	6.8	13.9	19.1
SL	0.1	1.7	1.8	16.9	3.3	0.8	3.2	5.7
SW	0.4	1.9	2.3	14.0	4.1	1.6	3.7	16.1
ST	0.1	0.8	0.9	5.7	3.9	1.1	3.7	8.6

δ^2^g, genotypic variance; δ^2^e, environmental variance; δ^2^p, phenotypic variance; GM, grand mean; PCV, phenotypic coefficient of variation (%); GCV, genotypic coefficient of variation (%); ECV, environmental coefficient of variation (%); H^2^, broad-sense heritability.

**Table 8:** Genetic variability among *S. madagascariensis* morphotypes for seventeen simple sequence repeat markers.

Locus	Dye	Primer Sequences (5′–3′) F: Forward; R: Reverse	SSR Sequence	AS	AN	MAF	GD	Ho	He	PIC	HWE	Null Allele
Ssp_1_	FAM	F: TGATGCAATGGATGTGTGCTAT	(ATTT)^6	144	5	0.85	0.27	0.89	0.64	0.26	NS	-
		R: TGAAGACGGCAATGCGAACC										
Ssp_2	ATTO532	F: TCGGAATACTACGGGCCACC	(AAAT)^5	199	4	0.85	0.27	0.89	0.64	0.25	NS	-
		R: TCCCTTCCAACCCTTCAATAAC										
Ssp_3	ATTO565	F: GTTGAGTTTGGACATTTGAAGGG	(AAAT)^5	203	1	1.00	0.00	0.00	0.00	0.00	Mono	N/A
		R: GAGCTAAGGTTCTGTGGCGG										
Ssp_4	FAM	F: ACCTCGTCACAATTACATATGCC	(ATTT)^6	165	7	0.33	0.76	0.30	0.78	0.72	**	0.45
		R: TTTAGTTGGCACTGGGGTCC										
Ssp_5	ATTO532	F: GGTAATTGGGCACGCTCTATG	(ATTT)^5	222	8	0.44	0.73	0.52	0.83	0.70	*	0.23
		R: ACTCCAACATAAGCCACTTTGC										
Ssp_6	FAM	F: GCCAGACAAGTTTCCCTCGG	(ATTT)^6	239	5	0.74	0.43	0.78	0.68	0.40	NS	-
		R: CCCGCGCTCAATGCTCTTAC										
Ssp_7	FAM	F: TCTTTGCTTTCTTCCTCGAAAGG	(ATTT)^5	281	7	0.56	0.65	0.67	0.82	0.63	NS	-
		R: GTATGATAGGTTCCACACGGC										
Ssp_8	ATTO532	F: GCCTATGGCAAGCAATGTATTC	(AACT)^7	285	2	0.96	0.07	0.00	0.07	0.07	*	0.06
		R: CCTTGAGTTCCAAGCTGCAC										
Ssp_9	ATTO550	F: CTGGACTGTCTTCTCGGGTTC	(AAAT)^5	288	5	0.74	0.43	0.04	0.41	0.40	**	0.38
		R: CAATTGCCAGTAACCGTGTAGG										
Ssp_10	ATTO550	F: GACATACAAATAGAAGCACTGG	(ATTT)^5	181	1	1.00	0.00	0.00	0.00	0.00	Mono	N/A
		R: CATGAGGGAAACCCACCCTG										
Ssp_11	ATTO565	F: ATTCTGGTCCCGTCACTGCC	(ATGC)^5	314	3	0.89	0.20	0.96	0.54	0.19	NS	-
		R: CTTCGGGTGCCAAAGTTCAC										
Ssp_12	FAM	F: TGCCTACTAACTAGCGTGAGG	(AAAT)^7	355	5	0.85	0.27	0.93	0.64	0.26	NS	-
		R: AGCCAGCGAATTGTGTTATCC										
Ssp_13	ATTO550	F: TCTATGTTGGAAATGCGCACG	(AATT)^5	140	4	0.89	0.21	0.00	0.21	0.20	**	0.19
		R: CATTGCACACAAAGCTACCTG										
Ssp_14	ATTO550	F: GTTGGGGGTTAAACATTCAGC	(ATTT)^5	248	4	0.89	0.21	0.00	0.21	0.20	**	0.19
		R: CACTTTTATGCTCCCGTGTCC										
Ssp_15	FAM	F: CAAGGTTTCGCCGAGCTGC	(AAAT)^6	377	9	0.44	0.74	0.00	0.75	0.71	**	0.48
		R: CTTGGAGTCCCAAGAAGCCG										
Ssp_16	ATTO550	F: AGAGTCAAGGAGGTTAGTGGC	(AAAT)^5	386	4	0.67	0.50	0.26	0.51	0.45	NS	-
		R: GATTCTCCCTTGAGTTCTAATGC										
Ssp_19	ATTO532	F: GATGGAGAGCCCAATGCAAG	(ATTT)^6	330	3	0.93	0.14	0.00	0.14	0.14	**	0.13
		R: GCTGTGAATTGTTAAAGGTCAAC										

F—forward marker; R—the reverse marker; AS—allele size; S—sample size; AN—allele number; MAF—major allele frequency; GD—genetic diversity; Ho—observed heterozygosity; He—expected heterozygosity; PIC—polymorphic information content; HWE—Hardy–Weinberg equilibrium; Mono—monomorphic locus; NS—Not significant; *—*p* < 0.05; **—*p* < 0.01.

## Data Availability

The data will be available from the authors on request.

## Supplementary Materials

The following supporting information can be downloaded at: https://www.mdpi.com/article/10.3390/genes17070732/s1. Table S1: Global Positioning of *S. madagascariensis* morphotypes at Bonamanzi Game Reserve. Table S2: Nei’s genetic distance of *S. madagascariensis* morphotypes using seventeen SSR markers.
