# Supplementary material for: Morphological and Genetic Variation in Strychnos madgascariensis Poir (Loganiaceae) at Bonamanzi Game Reserve, KwaZulu-Natal, South Africa
Source: Genes (Basel). 2026 Jun 24;17(7):732. doi: 10.3390/genes17070732 (PMC13407658; doi:10.3390/genes17070732)
Supplement: Supplementary file 1 [file genes-17-00732-s001.zip › genes-4363762-supplementary.pdf]

Table S1. Global Positioning of *S. madagascariensis* morphotypes at Bonamanzi Game Reserve

**Morphotype**	**Plant traits**				**Global Positioning System**		
	**Leaf colour**	**Leaf shape**	**Leaf hairiness**	**Leaf size**	**Latitude**	**Longitude**	**Altitude**
GEH1	Green	Elongated	Hairy	Small	-28.06193	32.29174	59
GEH2	Green	Elongated	Hairy	Medium	-28.05520	32.29578	60
GElH1	Green	Elongated	Less hairy	Small	-28.06649	32.28996	66
GElH2	Green	Elongated	Less hairy	Medium	-28.06943	32.28876	67
GElH3	Green	Elongated	Less hairy	Big	-28.05287	32.29652	57
GRH2	Green	Round	Hairy	Medium	-28.06930	32.28868	66
GRH3	Green	Round	Hairy	Big	-28.06941	32.28890	70
GRlH1	Green	Round	Less hairy	Small	-28.05033	32.29742	61
GRlH2	Green	Round	Less hairy	Medium	-28.05105	32.29705	63
GRlH3	Green	Round	Less hairy	Big	-28.05114	32.29707	59
GyEH1	Grey	Elongated	Hairy	Small	-28.05167	32.29698	60
GyEH2	Grey	Elongated	Hairy	Medium	-28.05499	32.29598	60
GyElH1	Grey	Elongated	Less hairy	Small	-28.05169	32.29690	61
GyElH2	Grey	Elongated	Less hairy	Medium	-28.05172	32.29690	61
GyEvH1	Grey	Elongated	Very hairy	Small	-28.06870	32.28922	63
GyEvH2	Grey	Elongated	Very hairy	Medium	-28.05066	32.29694	67
GyEvH3	Grey	Elongated	Very hairy	Big	-28.05509	32.29583	58
GyRH1	Grey	Round	Hairy	Small	-28.06934	32.28866	64
GyRH2	Grey	Round	Hairy	Medium	-28.05040	32.29754	64
GyRlH1	Grey	Round	Less hairy	Small	-28.05072	32.29756	62
GyRlH2	Grey	Round	Less hairy	Medium	-28.06619	32.29001	64
lGEH1	Light green	Elongated	Hairy	Small	-28.06918	32.28928	69
lGEH2	Light green	Elongated	Hairy	Medium	-28.05189	32.29686	62
lGElH1	Light green	Elongated	Less hairy	Small	-28.06820	32.28959	64
lGElH2	Light green	Elongated	Less hairy	Medium	-28.06788	32.28955	66
lGRH2	Light green	Round	Hairy	Medium	-28.05031	32.29760	66
lGRlH1	Light green	Round	Less hairy	Small	-28.05064	32.29742	62

Table S2. Nei's genetic distance of *S. madagascariensis* morphotypes using seventeen SSR markers

Morphotypes	GEH1	GEH2	GElH1	GElH2	GElH3	GRH2	GRH3	GRlH1	GRlH2	GRlH3	GyEH1	GyEH2	GyElH1	GyElH2
GEH1	-													
GEH2	1.33	–												
GElH1	0.75	1.04	–											
GElH2	0.89	1.04	0.23	–										
GElH3	0.75	1.04	0.19	0.23	–									
GRH2	0.75	1.04	0.19	0.16	0.13	–								
GRH3	0.69	1.04	0.19	0.16	0.19	0.19	–							
GRlH1	0.89	1.04	0.31	0.27	0.31	0.31	0.16	–						
GRlH2	0.82	1.04	0.35	0.31	0.35	0.35	0.19	0.31	–					
GRlH3	0.69	1.04	0.19	0.19	0.19	0.19	0.06	0.16	0.27	–				
GyEH1	0.82	1.04	0.23	0.31	0.31	0.31	0.23	0.35	0.39	0.16	–			
GyEH2	0.75	1.04	0.13	0.09	0.13	0.06	0.13	0.23	0.35	0.13	0.23	–		
GyElH1	0.82	1.04	0.19	0.16	0.19	0.19	0.06	0.23	0.27	0.13	0.23	0.13	–	
GyElH2	0.96	1.22	0.35	0.23	0.44	0.44	0.19	0.35	0.27	0.27	0.39	0.35	0.27	–
GyEvH1	0.58	1.04	0.19	0.27	0.27	0.27	0.13	0.23	0.27	0.13	0.23	0.19	0.19	0.27
GyEvH2	0.69	1.04	0.19	0.16	0.19	0.19	–	0.16	0.19	0.06	0.23	0.13	0.06	0.19
GyEvH3	1.04	0.58	0.53	0.44	0.64	0.64	0.44	0.44	0.53	0.44	0.53	0.53	0.53	0.44
GyRH1	1.04	0.96	0.75	0.89	0.75	0.75	0.75	0.96	0.69	0.64	0.64	0.75	0.75	0.89
GyRH2	0.53	1.04	0.35	0.44	0.35	0.35	0.35	0.48	0.44	0.27	0.31	0.35	0.35	0.53
GyRlH1	1.04	1.45	0.82	1.04	0.96	0.96	0.96	1.04	1.33	0.96	0.96	0.82	0.96	1.33
GyRlH2	0.75	1.04	0.19	0.16	0.13	–	0.19	0.31	0.35	0.19	0.31	0.06	0.19	0.44
lGEH1	0.69	1.04	0.19	0.16	0.19	0.19	–	0.16	0.19	0.06	0.23	0.13	0.06	0.19
lGEH2	0.82	0.89	0.44	0.31	0.44	0.44	0.27	0.44	0.13	0.35	0.44	0.44	0.35	0.27
lGElH1	0.69	1.04	0.19	0.23	0.19	0.06	0.27	0.39	0.35	0.27	0.31	0.13	0.27	0.44
lGElH2	0.48	1.04	0.27	0.31	0.27	0.19	0.35	0.48	0.44	0.35	0.39	0.19	0.35	0.53
lGRH2	0.75	1.22	0.31	0.13	0.31	0.31	0.16	0.27	0.31	0.19	0.39	0.23	0.23	0.23
lGRlH1	0.75	1.22	0.53	0.44	0.53	0.53	0.53	0.64	0.53	0.44	0.48	0.53	0.53	0.44

Table S2. Nei's genetic distance of *S. madagascariensis* morphotypes using seventeen SSR markers (continued)

Morphotypes	GyEvH1	GyEvH2	GyEvH3	GyRH1	GyRH2	GyRlH1	GyRlH2	lGEH1	lGEH2	lGElH1	lGElH2	lGRH2	lGRlH1
GEH1													
GEH2													
GElH1													
GElH2													
GElH3													
GRH2													
GRH3													
GRlH1													
GRlH2													
GRlH3													
GyEH1													
GyEH2													
GyElH1													
GyElH2													
GyEvH1	–												
GyEvH2	0.13	–											
GyEvH3	0.39	0.44	–										
GyRH1	0.75	0.75	0.75	–									
GyRH2	0.27	0.35	0.69	0.69	–								
GyRlH1	0.82	0.96	0.96	1.04	0.96	–							
GyRlH2	0.27	0.19	0.64	0.75	0.35	0.96	–						
lGEH1	0.13	–	0.44	0.75	0.35	0.96	0.19	–					
lGEH2	0.35	0.27	0.35	0.58	0.53	1.22	0.44	0.27	–				
lGElH1	0.23	0.27	0.58	0.75	0.31	0.89	0.06	0.27	0.44	–			
lGElH2	0.27	0.35	0.69	0.82	0.13	0.82	0.19	0.35	0.53	0.16	–		
lGRH2	0.27	0.16	0.44	1.04	0.53	1.04	0.31	0.16	0.31	0.39	0.48	–	
lGRlH1	0.44	0.53	0.69	0.82	0.13	1.33	0.53	0.53	0.53	0.48	0.27	0.53	–

– Represents a value of 0.00. Morphotypes are described in Table 1
